# Red Vulcanite as a Base for Artificial Teeth

**Published:** 1863-10

**Authors:** Adino. B. Hall

**Affiliations:** Boston


Red Vulcanite as a Base for Artificial Teeth.—*Messrs. Editors:* A correspondent of the London *Lancet* called the attention of the profession, some time ago, to the use of "red vulcanite," employed by dentists as the basis of artificial teeth. In alluding to the effects of this new material, he says:—

"I have at the present time a patient under my care who was supplied, some nine months since, with a set of teeth for the upper jaw, based on 'red vulcanite,' who has been declining in health and spirits ever since he has worn them. From being a strong, healthy, muscular and robust man, he has been losing flesh rapidly, with loss of appetite, sickness, nausea, flatulence, gastric irritation, fœtid breath, vertigo, diarrhœa, etc., apparently without any assignable cause. Seeing that he wore artificial teeth, I begged to be allowed to look at them, and it occurred to me whether there might not be something prejudicial in the *coloring* matter of the 'vulcanite,' which was keeping up his somewhat anomalous symptoms. I requested him to leave them off for a month (much against his inclination, as he never thought about his teeth having anything to do with his indisposition), and a marked improvement in all of his symptoms followed. He has since resumed them, with a like result as before."

Subsequently, another correspondent, Dr. H. C. Roods, referred to the same subject; and in some remarks, on the influence of the *coloring* matter (bisulphuret of mercury) of "vulcanite" upon health, he states that a relative of his, whose previous status of health had always been good, after wearing teeth prepared from this material for some months, began to complain of diarrhœa and an irritable state of the bowels, which persisted, notwithstanding the patient was put upon a well-regulated diet, and was subjected to several remedial agents. The thought occurred to the doctor that "vulcanite" might be the disturbing cause, inasmuch as mercurials of any kind, in the smallest doses, always produced, in this lady, great irritability of the alimentary canal.

In this community the same question has arisen—whether or not the general use of vulcanized rubber, as a basis for artificial teeth, is having a deleterious effect upon the health of those wearing it constantly, in contact with the glandular secretions of the mouth. Some cases have been verbally reported, in which there are grave suspicions that this dental material is producing an injurious influence upon the system. A case was recently related to me, of the sudden death of a man who wore a whole set of teeth on rubber. A *post-mortem* examination did not reveal any noticeable cause of death. It is well known that many persons are extremely susceptible to medicinal agents, however minute the quantity. The same holds true in regard to both mineral and vegetable poisons, which are often introduced into the system so insidiously that the nature and cause of declining health are not known till too late to rescue the unsuspecting victim from a premature death. "Although it appears improbable," says the *Lancet*, "that a mercurial preparation, combined with an impermeable substance like vulcanite, should escape therefrom in sufficient quantity to affect the system, still facts may prove stronger than probabilities in the matter; and although many persons wear the material without prejudice and with great satisfaction, occasionally a party peculiarly susceptible to the influence of mercury may possibly suffer from the coloring matter, and if so, it would be desirable to select some other than a mercurial pigment for tinting the vulcanite, or coraline, as it is termed."

This subject is one of some importance, and from the well known reputation of the leading dentists of our city, we do not believe, for a moment, that they would knowingly countenance the use of any material, in their profession, that would produce injurious consequences upon the animal economy.

As physicians are the conservators of the public health, it is right and just that they should watch with careful vigilance any and every cause that may be productive of disease.

If these few thoughts shall serve to call the attention of the profession to the subject matter, in order that in cases where vulcanized rubber is used, and any abnormal consequences follow, the relation of the one to the other may be appreciated, then the object of the writer will have been answered. ADINO B. HALL.

*Boston, September 24th,* 1863.

